# Range-wide phylogeographic structure of the vernal pool fairy shrimp (*Branchinecta lynchi*)

**DOI:** 10.1371/journal.pone.0176266

**Published:** 2017-05-04

**Authors:** Kristy Deiner, Joshua M. Hull, Bernie May

**Affiliations:** 1 Department of Animal Science, University of California Davis, Davis, California, United States of America; 2 Sacramento Fish and Wildlife Office, U.S. Fish and Wildlife Service, Sacramento, California, United States of America; National Cheng Kung University, TAIWAN

## Abstract

Wetland habitats across the world are experiencing rapid modification and loss due to accelerating habitat conversion. Impacts to wetland habitats are particularly acute in California where up to 90% of wetland habitats have been modified or lost. Vernal pool ecosystems have therefore undergone a dramatic loss in habitat and along with them an entire endemic fauna is under threat of extinction. Recent efforts to conserve vernal pool habitat and associated species have involved restoration and creation of vernal pools as well as translocations of threatened species. The vernal pool fairy shrimp, *Branchinecta lynchi*, is one of several endemic and federally listed species being targeted for translocations. To guide reintroduction and conservation, detailed information on range-wide population structure and diversity is needed. We collected genetic data from two mitochondrial genes throughout the known extant range of *B*. *lynchi* to elucidate population structure and diversity of the species. We found support for phylogeographic structure throughout the range of *B*. *lynch* associated with isolated watersheds and vernal pool regions previously identified in the recovery plan for the species. The underlying mechanisms responsible for this broad pattern of genetic structure have yet to be identified. However, the evidence of only a few haplotypes being shared across the species range and patterns of isolation by distance within vernal pool regions suggests dispersal limitation may play a role. These results stress that conservation programs, at a minimum, should consider using individuals from regional populations as sources for reintroductions to maintain historical patterns of genetic differentiation. Additionally, because genetic structure is associated with vernal pool regions which are based on local hydrology and geology, translocations should proceed considering the distance between donor and recipient sites.

## Introduction

Wetland ecosystems in America have experienced greater than 50% declines in extent since the 1800s [1] and California has experienced even greater ongoing declines where up to 90% of historical wetland habitat has been lost since 1850 [2–4]. An assemblage of unique vernal pool habitats are among the California wetland habitats experiencing continuing declines. California’s vernal pool wetland ecosystems are seasonally flooded, island-like ecosystems that fill in the winter and dry in spring and summer. They include a diversity of habitats that support a distinct assemblage of endemic and specialist species [5–7]. Vernal pools and their supporting uplands are among the most at risk habitats in California with an average loss of over 2430 hectares each year since 2005 [8]. Habitat loss is mainly due to a combination of land-use change through urbanization and agricultural conversion [8, 9]. Consequently, many vernal pool endemic species are of conservation concern with over 20 of these listed as threatened or endangered under the federal Endangered Species Act, including several vernal pool branchiopods (Anostraca and Notostraca) [9].

California’s vernal pool wetlands have been grouped into 17 distinct geographic regions based on species endemic to each region as well as geomorphology and underlying soils [4]. These vernal pool regions have subsequently formed the basis for understanding broad ecological patterns among California’s vernal pools and have guided ongoing conservation efforts including the Recovery Plan for Vernal Pool Ecosystems of California and Southern Oregon (Recovery Plan) [9]. Following publication of the Recovery Plan [9], vernal pool conservation efforts have focused on habitat protection and restoration activities have been initiated to address and ameliorate historical declines in the distribution and abundance of vernal pool ecosystems [10]. These actions alone, however, may not be sufficient to prevent local extirpations and loss of genetic diversity because vernal pool branchiopods in fragmented landscapes can have limited dispersal capabilities [11]. Due to unpredictable inter-annual variation in rainfall, in which some pools may not fill every year, vernal pool Branchiopods display a bet-hedging life history strategy in which cysts remain dormant in vernal pool sediments until breeding conditions are favorable [12]. Because the adult life stage is not visible in every year, vernal pool Branchiopods may be particularly sensitive to habitat conversion [13] and once eliminated from a local area their limited dispersal ability may prevent natural re-colonization of restored habitat limiting the efficacy of vernal pool restoration in absence of translocations as a conservation measure. To address this challenge, the Recovery Plan calls for implementation of reintroduction programs informed by genetic data to restore extirpated populations [9].

*Branchinecta lynchi* (Anostraca: Branchinectidae, Eng, Belk & Eriksen, common name vernal pool fairy shrimp) [6] is one of several federally listed branchiopods occurring in vernal pool ecosystems of southern Oregon and California. *B*. *lynchi* has the largest current distribution of any of the listed Branchiopods ranging from Jackson County in southern Oregon south to Riverside County in southern California; however, within this range *B*. *lynchi* occurs locally in a small proportion of the vernal pools [10] and is considered to be more fragmented and isolated compared with pre-agricultural times. Because of its sparse and isolated distribution across a broad range, *B*. *lynchi* is particularly susceptible to loss of habitat and demographic disruption from landscape fragmentation and would therefore benefit from targeted reintroductions [9]. *B*. *lynchi* occurs in vernal pools that vary in size (from 0.02 to 10 hectares), elevation (10 m to 1,700 m), temperatures (4.5 to 23°C), and are rarely found to co-occur with other fairy shrimp species [6]. It is preyed upon by the vernal pool tadpool shrimp (*Lepidurus packardi*) and the diet of vernal pool fairy shrimp largely consists of algae, bacteria, protozoa, rotifers and detritus [9]. Thus, the species inhabits a large niche; however, no specific studies have been done to assess its specific habitat requirements [9]. Given this large niche, the opportunities to reintroduce the species in remaining and newly constructed vernal pools are promising. In addition to demographic benefits of reintroductions, preservation of divergent populations and their extant genetic diversity can benefit the species by preserving adaptive capacity and resilience to catastrophes and changing environmental conditions [14, 15].

To guide ongoing reintroduction efforts and ensure preservation of extant genetic diversity of *B*. *lynchi*, additional information describing phylogenetic relationships among vernal pool regions and distribution of genetic variation throughout the species’ range is needed. Although *B*. *lynchi* is limited in mobility, several dispersal mechanisms, including waterfowl, wind, and flood events can likely result in long-distance movements of resting branchiopod eggs [16–18]. Because of these many dispersal mechanisms, branchiopods have no general expected patterns of phylogenetic structure. In some instances, the limited intrinsic dispersal abilities of branchiopods have resulted in regional differentiation with some degree of localized gene flow [19, 20]. This pattern has been associated with migration through temporary surface water connections between vernal pools [11, 20, 21]. A similar pattern of differentiation has also been found in rare vernal pool plant and vertebrate populations [22, 23]. In contrast, populations of four *Limnadopsis* species display no population structure across large portions of central Australia, possibly due to dispersal by large populations of water birds [24, 25].

Within *B*. *lynchi*, Aguilar [26] found limited evidence of geographic differentiation, but unique haplotype diversity was found within sampled locations, suggesting conservation efforts should focus at regional scales. However, the finding of local unique diversity and limited divergence at larger scales seems contradictory. We therefore sought to examine a broader geographic sample of individuals collected from throughout the range of the species and increase the breadth of sequence data included. Unfortunately, the limited availability and utility of microsatellite loci in branchiopods [27, 28] precludes their use for a detailed examination of population structure in *B*. *lynchi*. In this study, we examined nearly 400 individuals and sequenced two mitochondrial (mtDNA) genes, cytochrome c oxidase I (COI) and the ribosomal RNA gene 16S to test for regional population structure and potential management units across the species range, as well as identify if long distance dispersal is common or uncommon across the range.

## Materials and methods

### Ethics statement

Because *B*. *lynchi* is listed on the by the federal government as a threatened species and is of conservation concern, we sought to minimize new collection of samples from extant populations and, to the extent possible, we utilized museum collections for the sampling throughout the species’ range. There was no ethics review required for use of specimens previously collected from museums and all collections from new individuals was conducted by individuals possessing permits issued by the Fish and Wildlife Service and were collected in strict accordance to the requirements under the federal Endangered Species Act. Permit numbers and individual are listed in the acknowledgments section.

### Sampling

**Fig 1:**
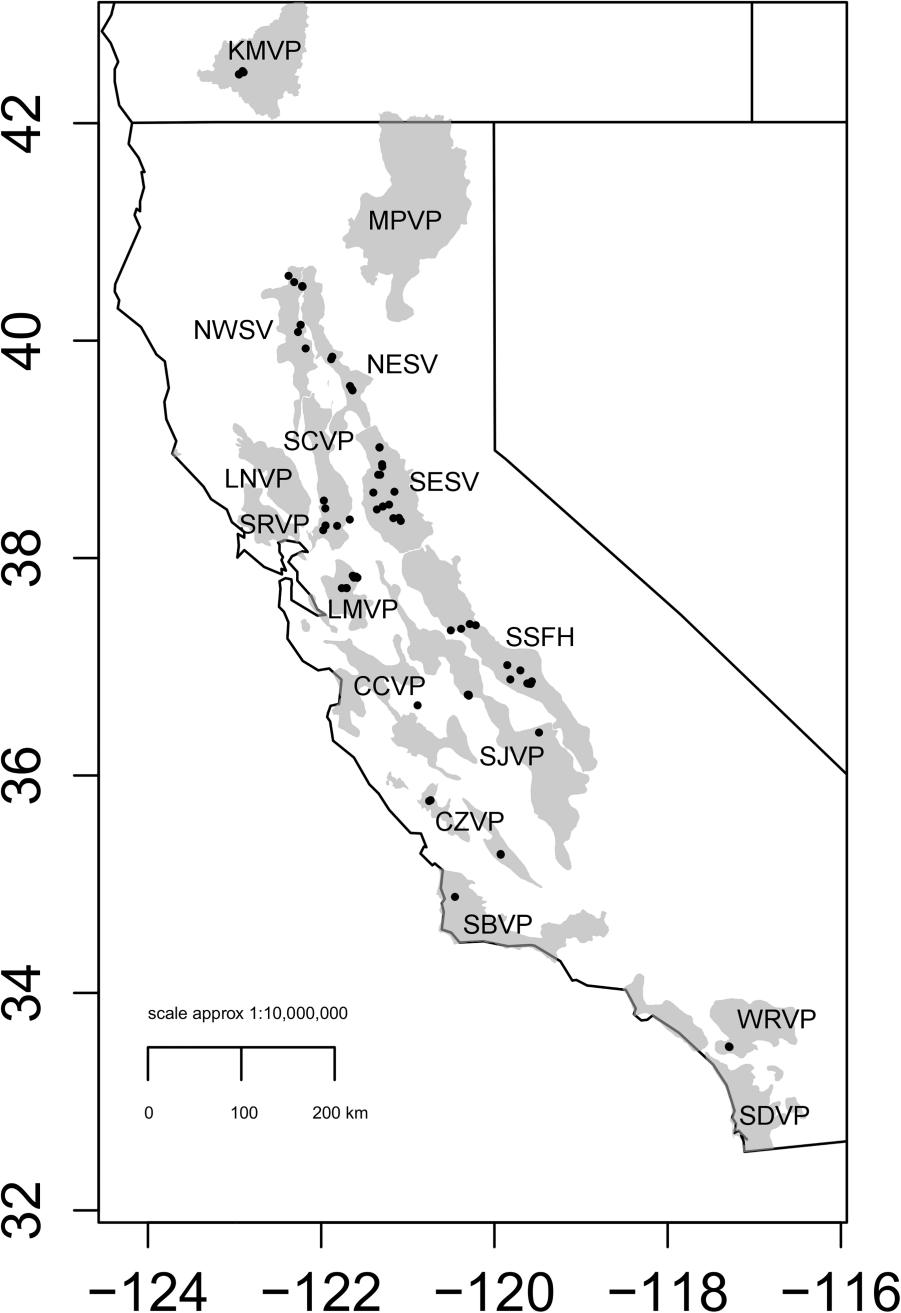
Map depicting in the extent of vernal pool regions throughout California (gray) and Southern Oregon and sampling localities (black dots) from which genetic data was generated for an analysis of phylogeographic structure and diversity for the species of *Branchinecta lynchi*. Vernal pool region abbreviations are as follows: Carrizo (CZVP), Central Coast (CCVP), Klamath Mountains (KMVP), Livermore (LMVP), Northeastern Sacramento Valley (NESV), Northwestern Sacramento Valley (NWVP), San Joaquin Valley (SJVP), Santa Barbara (SBVP), Solano-Colusa (SCVP), Southeastern Sacramento Valley (SESV), Southern Sierra Foothills (SSFH), and Western Riverside (WRVP). *B*. *lynchi* are not known from the Santa Rosa (SRVP), Lake-Napa (LNVP), Modoc Plateau (MPVP) or San Diego (SDVP) vernal pool regions in historical or contemporary samples and thus no sample locations were evaluated from these locations for this study.

The California Academy of Sciences holds a large collection of *B*. *lynchi* individuals (N = 3845; as of 2009 when sampling was initiated). From the collection, we were given permission to destructively subsample 483 specimens for genetic analysis (Table 1). Additionally, we were given permission to destructively subsample 17 specimens from the Santa Barbara vernal pool region from the Santa Barbara Museum of Natural History (Table 1). In geographic areas where the number of existing museum samples was insufficient for phylogeographic analysis, we received 196 specimens from extant populations in 2010 and 2011 collected by collaborators and from the U.S. Fish and Wildlife Service voucher specimen collection (Table 1). All extant samples were identified by morphological key prior to DNA extraction following Ericksen and Belk [29]. In total, we sampled 696 individuals to achieve near complete sample representation from throughout the species range (Fig 1). The vernal pool regions of San Joaquin (SJVP) and the Central Coast (CCVP) were limited by scarcity of individuals in museum collections and/or scarcity of remaining habitat from which to sample. Additionally, individuals were not sampled from existing vernal pools likely because of annual variability in emergence of the species during collection trips. Therefore, sample sizes for these regions were limited.

**Table 1:** Description of samples used to estimate phylogenetic diversity with COI and 16S in *Branchinecta lynchi* across the species range.

Vernal pool region	Abbreviation	number of vernal pools ^a^	N Total	N COI	N 16S	H COI	Haplotype names	H 16S	Haplotype names
Carrizo	CZVP	4	64	22	25	8	2–9	5	8–12
Central Coast	CCVP	1	3	0	3	0	na	1	**7**
Klamath Mountains	KMVP	4	34	10	13	6	10–15	1	13
Livermore	LMVP	10	45	25	45	7	16–22	4	**7**, 14–**16**
Northeastern Sacramento Valley	NESV	5	52	14	37	7	25–28, **29**, 30–31	6	17, **18**, 19–22
Northwestern Sacramento Valley	NWSV	11	117	55	82	18	23–24, 32–47	10	**7, 16**, 23, **24**, 25–27, **28**, 29–30
San Joaquin Valley	SJVP	4	10	2	8	2	75–76	3	**7**, 43–44
Santa Barbara	SBVP	1	17	0	10	0	na	5	**7, 18, 24, 31–32**
Solano-Colusa	SCVP	7	22	10	19	7	48–50, **51**, 52–54	5	**7, 18, 31**, 33–34
Southeastern Sacramento Valley	SESV	15	96	39	54	18	**51**, 55–72	12	**7, 18, 31–32,** 35–42
Southern Sierra Foothills	SSFH	27	210	40	66	27	**29**, 73–74, 77–101	18	**7, 15, 28,** 45–59
Western Riverside	WRVP	4	26	4	24	4	102–105	2	60–61
	Total	93	696	221	386	104^b^		55^b^	

N is number of individuals or sequences and H is the number of haplotypes and na stands for not applicable.

^a^ Number of vernal pools represents only pools for which we were able to obtain genetic data and not all sites sampled. Bolded numbers are the shared haplotypes between vernal pool regions.

^b ^Haplotypes are only counted once in total.

### DNA extraction, amplification and sequencing

DNA from the right ventral phyllopods of museum specimens or freshly collected specimens (N = 696, Table 1) was extracted using DNeasy Blood & Tissue Kits (Qiagen, Inc. Valencia, CA, USA) following the manufacturer’s recommended protocol except two elutions of 100 μL were performed with AE buffer that was warmed to 56°C. These phyllopods were utilized because they can be removed from a specimen and not substantially decrease the value of it for future morphological analysis and there is a high likelihood of concentration of mitochondria due to the physical use of these structures for swimming, feeding, and gas exchange. The use of such tissues rich in mitochondria can decrease the likelihood of psuedogene amplification which is common in crustaceans [30]. Extracted DNA was quantified using a Nanodrop spectrophotometer and diluted to 10 ng/μL using molecular grade DNA free water (Sigma-Aldrich, Co. LLC. St. Lewis, MO., USA). COI was amplified via polymerase chain reaction (PCR) using universal primers LCO1490 and HCO2198 [31]. For specimens that did not amplify with the universal COI primers, we used previously developed primers JyothiF-1489 for the forward and BrUniR-2042 or LYR2198 for the reverse [32] to produce a PCR product that was 423 bp. Additionally, for samples that still did not work for this fragment, we amplified a 135 bp fragment using a primer (COI-FS-intF, 5’—GATATGGCCTTYCCACGRCT—3’) we designed based on an alignment of sequences that had worked with the other primer sets above. We paired this newly developed primer with the previously designed universal primer for Branchinecta for amplification (BrUniR-2042) [32]. We designed new 16S primers for use in this study to amplify a 358 bp region (16S-FintE, 5’- AGGGCCGTGGTATTTTAACC-3’ and 16S-RintE, 5’- ATCCCTGAACCAACATCGAG—3’) using Primer 3 with default settings in version 4.0.0 [33, 34].

Each PCR contained TAQ buffer (1x) (Roche, Inc., Basel CH), BSA (1x), magnesium chloride (2mMol), dNTPs (0.20μMol), 0.05 units Faststart TAQ (Roche, Inc., Basel CH), 1 μL of DNA template (10 ng/μL), forward and reverse primers (0.50 μMol), and molecular grade water in a total reaction volume of 10 μL. Reported concentrations are in the final volume for the PCR. PCR for all primer sets involved a 4-minute denaturing at 94°C before thermocycling for 35 cycles. The thermocycling profile was 94°C denaturing for 30 s, annealing for 30 s, and 72°C extension for 1 minute, followed by a final 7-minute extension at 72°C. Annealing temperatures for COI primers sets were: 48°C for primer set LCO1490 and HCO2198 and primer set CO1-FS-intF and BrUniR-2042 and 50°C for primers JyothiF-1489 for the forward and BrUniR-2042 or LYR2198 for the reverse [32]. The annealing temperature for the 16S primer set was 53°C based on the average T_m_ of the two primers and then subtracting 5°C. PCR success was assessed on a 1.2% agarose gel stained with Gelstar (GE Healthcare, Waukesha, WI, USA). PCR products were purified using the ExoSap-it kit (GE Healthcare, Waukesha, WI, USA) and then sequenced bi-directionally on an ABI 3130/3730 sequencer using di-deoxy chain termination chemistry with BIG Dye v3.1 (Applied Biosystems, Grand Island, NY, USA) following recommended Applied Biosystems protocols. Sequences from forward and reverse primers were aligned and edited with SEQUENCHER v4.9 (GeneCodes, Ann Arbor, MI, USA). A global alignment for each gene was exported from SEQUENCHER in a nexus format and used for phylogenetic analysis. Sequences generated for both loci have been deposited in GenBank (KY795336—KY795556 for COI and KY795557—KY795942 for 16S, S1 Table). Furthermore, we submitted eight sequences from a subset of specimens hosted in the collection at the California Academy of Sciences to the Barcode of Life Database (BOLD) to represent the full length molecular barcodes for the species (BOLD accession numbers BLYN001-14 to BLYN008-14).

### Utility of museum specimens for phylogeographic analysis

In order to determine the utility of museum specimens of *B*. *lynchi* for phylogeographic analysis, we defined success for PCR amplification as a PCR product of expected size visible on a gel. Sequencing success was defined as the generation of high quality sequences for both the forward and reverse directions from the PCR product, but did not necessarily take the final length of sequence into account.

### Phylogeographic structure and diversity analysis

Only specimens that produced the full length sequence for the standard barcode region of COI (658 bp of the 5’ end of COI) [35] were included in all analyses for this gene (N = 221, Table 1). For 16S, only specimens that produced the full-length sequence for the targeted 358 bp region were used in all analyses for this gene (N = 386, Table 1). Also, due to some evidence of psuedogene amplification, all heterozygous sequences were excluded from counts in Table 1 and from all analysis for both COI (N = 39) and 16S (N = 41) [30]. Genes could not be concatenated because specimens yielded more 16S sequences compared with COI. We compared a larger dataset of COI sequences (315 samples) cut to a length of 423 bp to match haplotypes previously published for this species [26]. However, analysis with the shorter fragment revealed that there was less phylogenetic signal caused by a decrease in the number of informative sites by 41.5% (exclusion of 37 of the 89 informative sites). We therefore included only the full targeted sequence length (i.e., 658 bp for COI and 358 bp for 16S) for each specimen and performed phylogenetic reconstruction separately on each gene because it has been shown that missing data for whole genes or large stretches within a gene likely biases phylogenetic analysis [36]. Before phylogenetic reconstruction, we removed redundant haplotypes for both genes using DNAsp v 5.10.01 [37]. We used jModelTest V0.1 [38, 39] to find the best-fit model for our mtDNA data using the Akaike Information Criteria. The general time reversible model with a gamma rate distribution and invariant sites parameter (GTR+I+G) was the best-fit model for both genes and was used in subsequent maximum likelihood searches. We searched for the best-fit tree with GARLI v2.0 [40] using the estimated maximum likelihood criterion. A bootstrap analysis of 9999 replications was performed in GARLI to assess support for nodes. Additionally, we performed a Bayesian phylogenetic analysis using MrBAYES v3.1.2 [41] with default parameters except the following, Lset nst = 6 rates = invgamma; mcmc ngen = 20000000; printfreq = 10000; samplefreq = 1000; nchains = 4. We used a burnin of 200,000 trees and removed these before calculation of the posterior probabilities for node support. We used a representative taxon from a closely related species (*Branchinecta lindahli*) as the outgroup for phylogenetic analysis with the COI gene and *B*. *coloradensis*, *B*. *oriena*, *B*. *constricta*, *and B*. *paludosa* for rooting the 16S gene phylogeny. Support for phylogenetically distinct groups within the species was assessed by bootstrap values greater than 65% and/or a posterior probability of 95 or greater for the Bayesian analysis.

Due to a lack of phylogenetic signal in the 16S gene, all remaining phylogeographic analyses were performed using only COI haplotypes except for the network analysis. COI haplotypes within vernal pool regions were compared to estimate the number of transitions (n_s_), transversions (n_v_), and substitutions (n_d_) as implemented in Arlequin v3.5. Mean pairwise differences among haplotypes (π) and haplotype diversity (h) within vernal pool regions were calculated using DNAsp v 5.10.01 [37]. We estimated pairwise Ф_st_ among vernal pool regions using Arlequin v3.5 [42]. Classical F_st_ estimates use haplotype frequencies for mtDNA and there were only two instances of a shared haplotype between vernal pool regions, we therefore estimated Фst with the Kimura 2-parameter (K2P) genetic distance as implemented in Arlequin v3.5. This estimate of Фst takes sequence differences among haplotypes into account [42]. Significance of pairwise Фst estimates were assessed by 1,000 permutations as implemented in Arlequin v3.5.

Qualitatively we evaluated patterns of phylogenetic clustering among *B*. *lynchi* COI and 16S haplotypes using a reduced median-joining network in network version 4.2.0.1 [43]. In these networks, star-like networks are frequently associated with recent population expansion while extensively structured networks are indicative of long-term stable populations. To test for patterns consistent with population expansion or recent bottlenecks within vernal pool regions quantitatively, we evaluated the neutrality tests of Fu’s F_S_ [44] and Tajima’s D [45] as implemented in Arlequin v3.5. A significant (p < 0.02) negative value of Fu’s F_S_ is caused by an excess in alleles and can be indicative of population expansion or genetic hitchhiking, whereas a significant and positive Fu’s F_S_ is caused by an allele deficiency that is indicative of a recent population bottleneck (or overdominant selection). Likewise, significant and negative Tajima’s D values are evidence of low frequency alleles relative to expectations and can be indicative of recent population expansion, but can also be purifying selection. Significant (*p* < 0.05) positive values can be interpreted as recent population bottlenecks or balancing selection. We additionally performed mismatch distribution analysis [46] that compares the observed allele frequencies to those estimated under the spatial expansion model assuming constant deme size as implemented in Arlequin v3.5. To evaluate the significance of the distribution and its departure from the spatial expansion model we calculated the distribution of the test statistic SSD (the sum of squared differences) between the observed and the estimated mismatch distribution by a bootstrap approach as implemented in Arlequin v3.5. If the observed distribution of allele frequencies is multimodal this indicates the population is at demographic equilibrium. If the observed distribution of allele frequencies is unimodal then this indicates recent demographic or range expansion. Congruence among these three estimates are interpreted as evidence of bottlenecked populations (i.e., significant and positive Fu’s F_S_ and Tajima’s D and multimodal mismatch distributions) or populations under demographic or range expansion (i.e., significant and negative Fu’s F_S_ and Tajima’s D and unimodal mismatch distributions).

**Fig 2:**
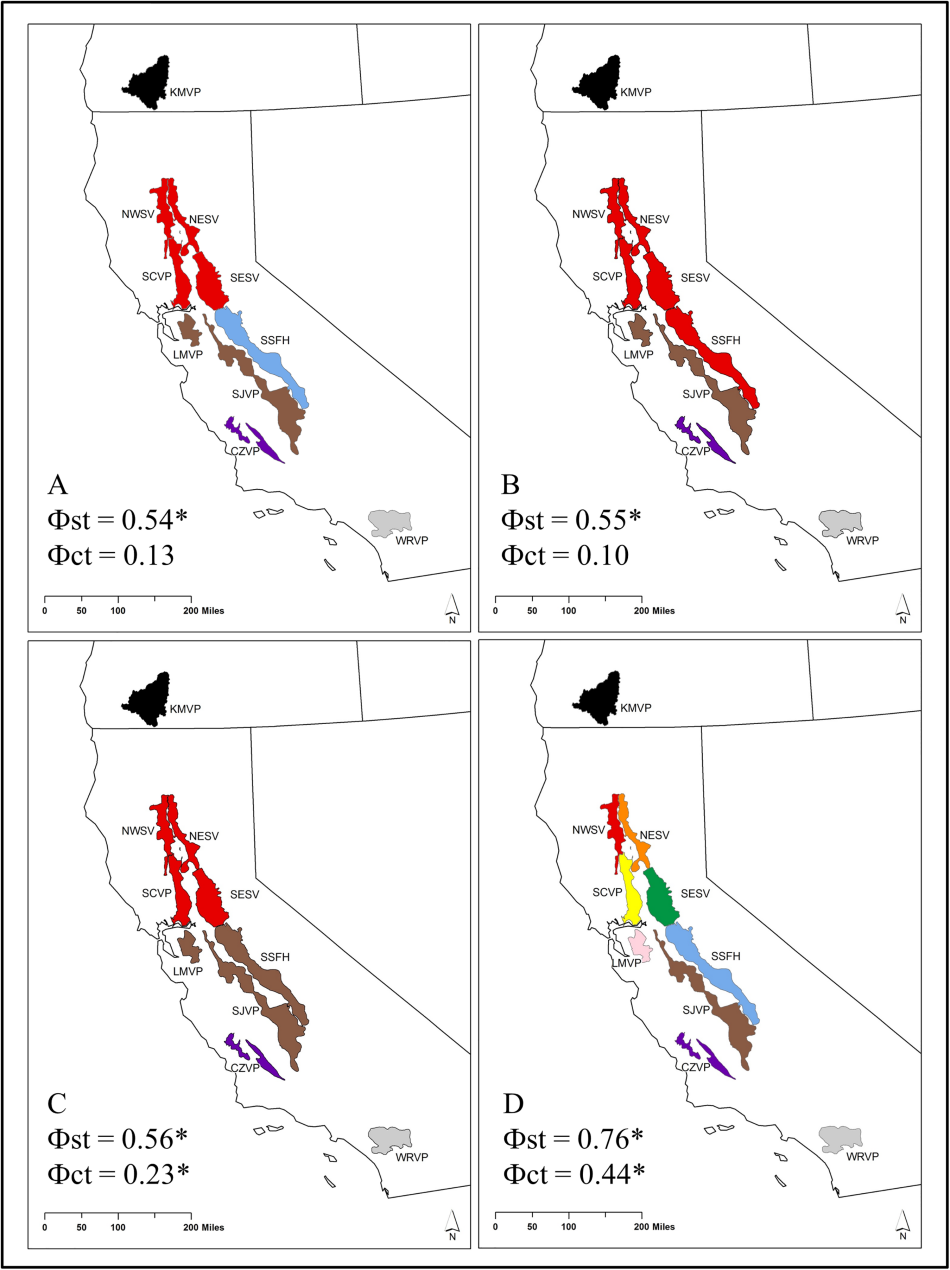
Putative isolated geographic regions that were tested for an analysis of molecular variance. See Table 1 for abbreviations for vernal pool regions indicated in colors in panel D. Larger watersheds are Klamath River in black, Sacramento River in red, the San Joaquin River in Brown, the Central Coast watersheds in purple, the Sothern Sierra Foothills watersheds in light blue, and the Southern coast watersheds in grey. A) Model 1 tested whether larger watersheds best explain the population genetic structure populations of the vernal pool fairy shrimp treating the Southern Sierra Foothills vernal pool region as an independent watershed. B) Model 2 tested whether larger watersheds best explain the population genetic structure populations of the vernal pool fairy shrimp treating the Southern Sierra Foothills vernal pool region as belonging to the Sacramento River watershed. C) Model 3 tested whether larger watersheds best explain the population genetic structure populations of the vernal pool fairy shrimp treating the Southern Sierra Foothills vernal pool region as belonging to the San Joaquin River watershed. D) Model 4 tested whether individual vernal pool regions best explain the population genetic structure of *Branchinecta lynchi*. Starred values of Φ_st_ and Φ_ct_ are significant at a *p*-value of 0.05 or less.

An analysis of molecular variance (AMOVA) was performed with only COI mtDNA pairwise estimates of Фst in Arlequin v3.5 [42] to test whether partitioning of genetic variance was significant among putative barriers to gene flow. Barriers to gene flow were hypothesized to be between watersheds as a major landscape features causing vicariant isolation among regions across the species’ range or vernal pool regions as established in the Recovery Plan [9]. Specimen collection locations were mapped and designated as belonging to either vernal pool regions or as belonging to independent watersheds using designations established by the Recovery Plan and shown in Fig 1 [9]. The major watersheds we hypothesized as putative barriers were the Sacramento River, the San Joaquin River, the Klamath River, the Central Coast and the South Coast (Fig 2). We tested four hypotheses based on these putative barriers given the uncertainty of the Sierra Nevada Foothill populations. Because haplotypes sampled from this vernal pool region had evidence of similar ancestry to that of many vernal pool regions (see results in [26]), it was unclear whether it should be treated as a separate population (model 1, light blue in Fig 2A) or as belonging with the Sacramento River (model 2, red in Fig 2B) or San Joaquin River (model 3, Fig 2C) drainages. Model 4 (Fig 2D) tested whether genetic variation could be best explained by structure between vernal pool regions as established in the Recovery Plan [9].

To test for dispersal limitation, we conducted an isolation by distance analysis within and between vernal pool regions. We estimated the pairwise geographic distance between sample locations in kilometers using QGIS v2.6 [47] and correlated this geographic distance matrix with a corrected pairwise K2P genetic distance matrix. K2P genetic distance was used in place of F_st_ because so few haplotypes were shared between vernal pool regions. Significance of isolation by distance correlations were determined by a Mantel test with 1,000 permutations as implemented in Arlequin v3.5 [42].

## Results

### Utility of museum specimens for phylogeographic analysis

**Fig 3:**
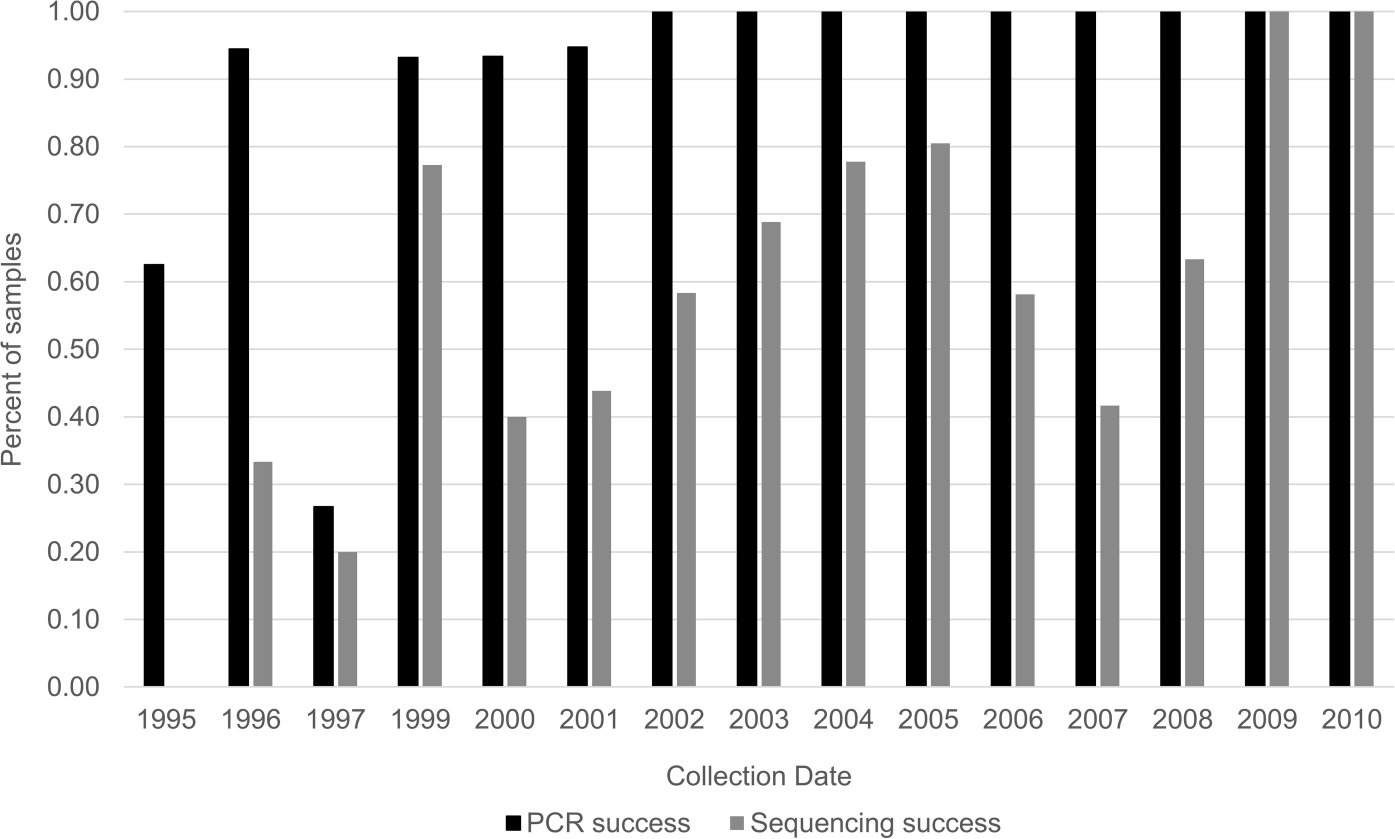
Depicts the negative relationship between the age of the *Branchinecta lynchi* specimen and its success for amplification and sequencing for two mitochondrial genes (COI and 16S).

We successfully amplifyed and sequenced the targeted gene regions for many museum specimens (Table 1). Success of both amplification and sequencing was negatively correlated with the age of the specimen with the oldest specimens producing the lowest amplification and sequencing success (Fig 3). Because of the difficulties with sequencing *mt*DNA due to the age of a specimen, of the 696 samples analyzed for COI, only 224 samples successfully produced COI sequences of 658 bp and only 386 samples produced 16S sequences of 358 bp for a total of 399 specimens producing sequence data for at least one gene (S1 Table).

### Phylogenetic structure and diversity recovered with COI

**Fig 4:**
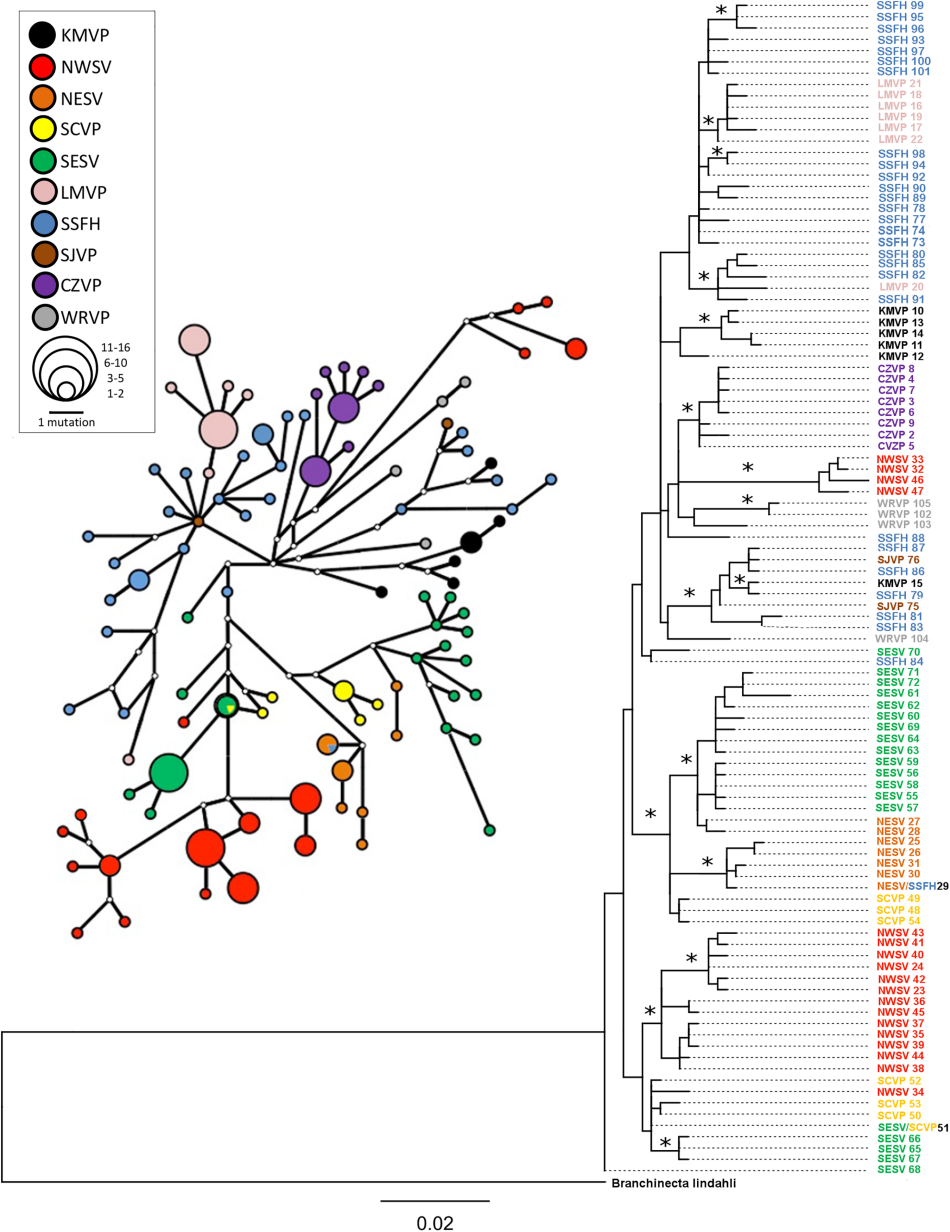
Depicts the evolutionary relationship of COI haplotypes across the species range and the network of their relatedness. Open circles in network are hypothesized but not sampled haplotypes. Abbreviations are as in Table 1. Asterisks on nodes of tree indicate that this group of haplotypes had a bootstrap support greater than 65% and/or Bayesian support of a posterior probability greater than 95 (see S1 and S2 Figs for all support values).

The initial morphological identification, the sequence similarity of the recovered sequences with previously published *B*. *lynchi* sequences, and monophyly of sequences with respect to the outgroups indicate that both the museum samples and those that were provided through the Fish and Wildlife Service belong solely to *B*. *lynchi*. Based on the maximum likelihood and Bayesian phylogenetic analyses of the 658 bp fragment of COI, we found significant phylogenetic structure for Carrizo and Klamath Mountains and limited sharing of haplotypes among vernal pool regions (Fig 4, S1 and S2 Figs). However, analysis does not include haplotypes from Central Coast and Santa Barbara vernal pool regions because we were unable to successfully obtain sequences from samples collected in these regions for the full-length dataset of 658 bp.

The AMOVA analysis overall supports that within and between group variance is maximized in the model where all vernal pool regions are treated as separate populations (Fig 2D). Pairwise Фst among vernal pool regions was significant for all comparisons except one and ranged between 0.14 and 0.80 (Table 2). The comparison that was not significant (SJVP to WRVP) was between the two vernal pool regions where we had very low samples sizes. The lowest Фst between two regions was between SCVP and SESV, one of two pairs of vernal pool regions for which we observed a shared haplotype (SCVP/SESV 51, Fig 4). One other haplotype was like those sampled from SSFH region but was sampled in KMVP region (KMVP 15, Fig 4). The KMVP haplotype 15 shows a high degree of similarity with haplotypes from the SSFH, but it differs by two base pairs from the most closely related haplotype SSFH 79 (Fig 4).

**Table 2:** Estimated pairwise differentiation (Фst) from the COI gene among vernal pool regions.

	SCVP	SESV	NESV	NWSV	SJVP	LMVP	SSFH	CZVP	KMVP	WRVP
SCVP	0.00									
SESV	0.14	0.00								
NESV	0.43	0.38	0.00							
NWSV	0.38	0.38	0.63	0.00						
SJVP	0.62	0.52	0.73	0.59	0.00					
LMVP	0.73	0.61	0.81	0.62	0.82	0.00				
SSFH	0.39	0.42	0.59	0.46	0.33	0.29	0.00			
CZVP	0.69	0.54	0.78	0.59	0.80	0.82	0.45	0.00		
KMVP	0.57	0.52	0.68	0.59	0.56	0.74	0.42	0.72	0.00	
WRVP	0.52	0.46	0.64	0.53	0.32^a^	0.74	0.33	0.66	0.51	0.00

Abbreviations are as in Table 1.

^a^ Value not significant at p = 0.05 or less.

The network analysis of haplotypes suggests some geographic structuring as indicated by the non-sharing of haplotypes among most regions and the close sequence similarity of haplotypes within each vernal pool region (Fig 4). No star-like pattern for the network was observed across the whole species range. The Carrizo Plains vernal pool region showed a similar pattern to that of the SSFH, but none of the values were significant. The Northeast Sacramento Valley vernal pool region had a mismatch distribution consistent with a recently bottlenecked population having a high and significant raggedness index and both Fu’s F_s_ and Tajima’s D were positive, however, these values were not significant (Table 3, S3 Fig). All other sites showed no significant evidence of recent population expansion or bottlenecks (Table 3).

**Table 3:** Estimated molecular diversity indexes for *Branchinecta lynchi* for vernal pool regions.

VPR	n_s_	n_v_	n_d_	*h*	*π*	D	F_S_	MMD	Rg	Rg p-value	SSD	SSD p-value
CZVP	10	0	10	0.81	0.003	-1.01	-2.08	Uni	0.06	0.49	0.02	0.41
KMVP	24	1	25	0.84	0.011	-0.85	1.30	Multi	0.14	0.40	0.07	0.28
LMVP	16	2	18	0.72	0.004	-1.57	0.13	Multi	0.09	0.65	0.02	0.56
NWSV	47	2	49	0.89	0.013	-0.70	-0.11	Multi	0.03	0.72	0.02	0.56
NESV	16	2	18	0.89	0.010	0.62	1.48	Multi	0.17	**0.02**	0.08	**0.03**
SCVP	13	1	14	0.91	0.009	1.01	-0.29	Multi	0.14	0.59	0.10	**0.04**
SESV	37	2	39	0.83	0.014	0.08	-1.59	Multi	0.04	0.73	0.02	0.48
SJVP	4	0	4	1.00	0.006	0.00	1.39	n/a^a^	n/a^a^	n/a^a^	n/a^a^	n/a^a^
SSFH	54	8	62	0.98	0.016	-1.12	**-8.75**	Uni	0.01	0.61	0.01	0.62
WRVP	19	1	20	1.00	0.017	0.24	0.46	Multi	0.22	0.78	0.11	0.31

n_s_ = number of transitions; n_v_ = number of transversions; n_d_ = number of substitutions; *h* = haplotype diversity; *π =* nucleotide diversity; and indicators of demographic or spatial expansion (Tajima's D and Fu's F_S_). Bolded values for D and F_S_ are significant at the *p*<0.01. MMD is the shape of the mismatch distribution (unimodal or multimodal); R_g_ is the Harpending's raggedness index.

^a^ Sample size for SJVP was too low to estimate MMD.

Among the AMOVA models tested for placement of the SSFH vernal pool region, Фst and Фct were both significant only when SSFH was grouped with other vernal pool regions in the watershed of the San Joaquin River (Fig 2C). However, the pairwaise Фst analysis suggests that the SSFH vernal pool region is the least differentiated from any other vernal pool region with the lowest average among-region Фst of 0.38 (Table 2). The Southern Sierra Foothills vernal pool region had closely related haplotypes to many vernal pool regions (Fig 4) and had high haplotype and nucleotide diversity (Table 3). Additionally, the neutrality tests indicate that only the Southern Sierra Foothills vernal pool region has strong support for a recent expanding population as indicated by the negative and significant Fu’s F_s_ (Table 3). Tajima’s D was negative; however, it was not significant, and the mismatch distribution was unimodal (Table 3, S3 Fig).

**Fig 5:**
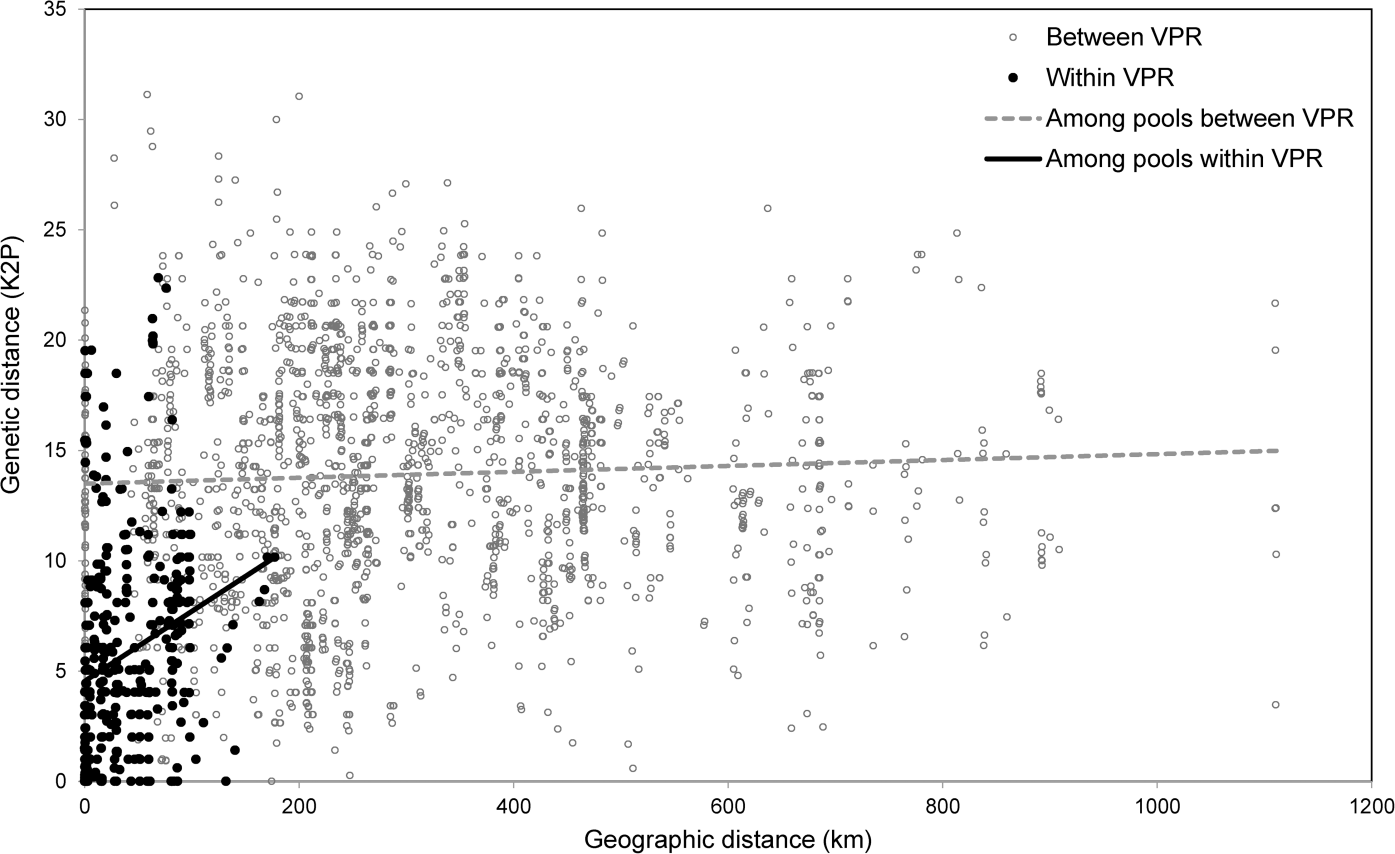
Depicts a significant pattern of isolation by distance across the species’ range between geographic distance in kilometers and genetic distance (corrected K2P; r = 0.278, p = 0.000). Black dots are comparisons within a vernal pool region and grey circles are comparisons between vernal pool regions. Lines are for illustration purpose only to show the direction of the correlation of within vernal pool region (solid line) versus between vernal pool regions (dashed line).

**Fig 6:**
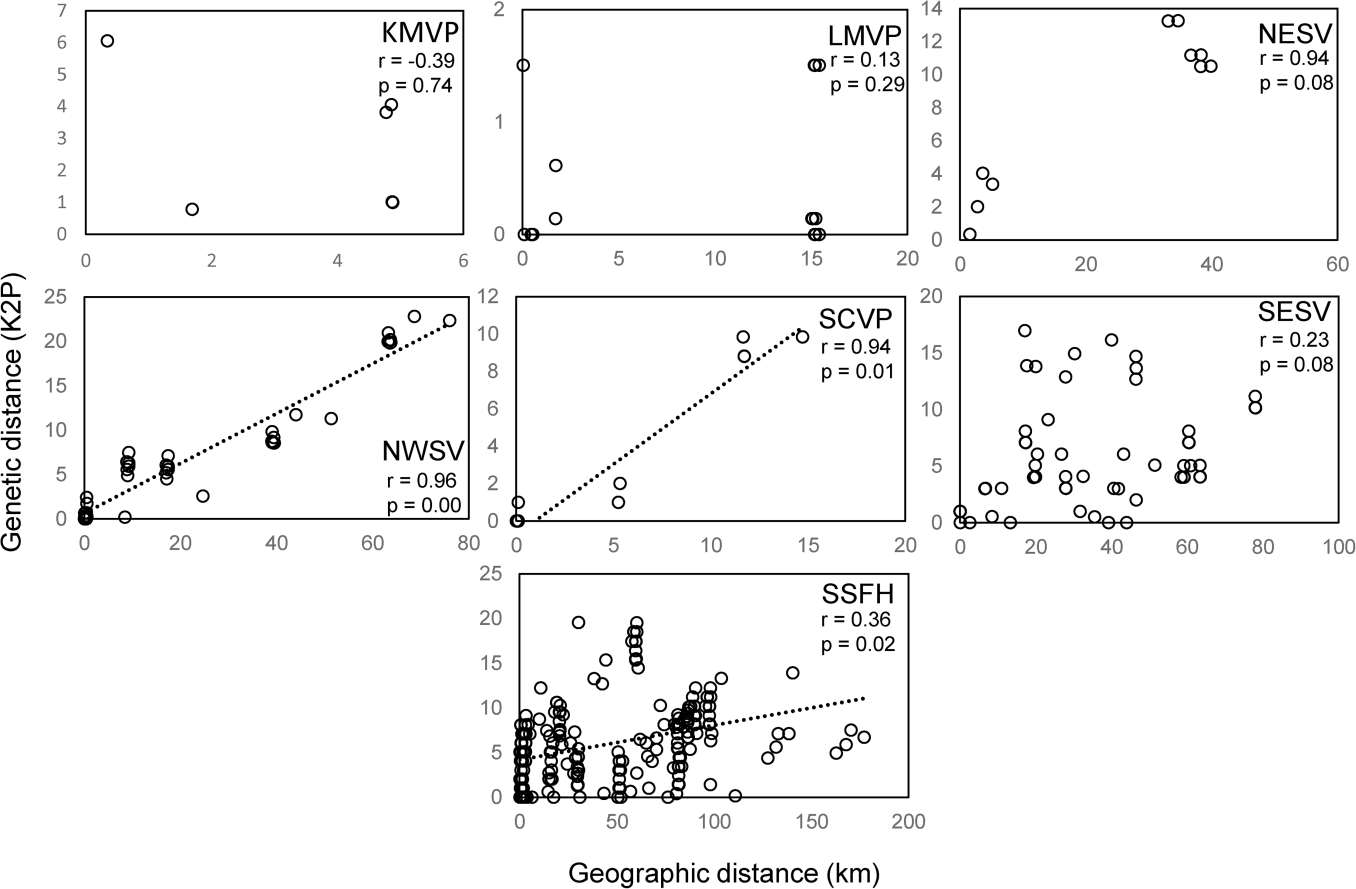
Depicts the patterns of isolation by distance within each vernal pool region between geographic distance in kilometers and genetic distance estimated with corrected K2P. CZVP, SJVP and WRVP regions were not analyzed due to low sample size.

Overall, correlation tests indicate a significant pattern of isolation by distance across all sites (R = 0.278, p = 0.000, Fig 5). However, the correlation coefficient was low (R = 0.278) and this pattern only explained 10% of the amount of genetic differentiation explained by geographic distance. When comparing pairwise geographic distance to genetic distances between sites sampled within a vernal pool region to sites in other vernal pool regions we found no relationship (dotted line Fig 5). However, we found a positive slope for many within vernal pool region comparisons (solid line Fig 5). Analysis of isolation by distance within each vernal pool region revealed positive and significant relationships for some regions, but no general pattern in geographic distance was observed (Fig 6). CZVP, SJVP and WRVP regions were not analyzed due to low sample size.

### Phylogenetic structure and diversity recovered with 16S

We successfully sequenced more individuals from the museum collections for the 16S gene, but the diversity was low (only 55 haplotypes, Table 1) and a single haplotype (16S haplotype 7, Table 1) was found across the species range in 138 out of the 386 sequenced individuals. The dominance of this single haplotype resulted in no strong phylogenetic signature across the species’ range, however we found that few haplotypes were shared among vernal pool regions (S4 Fig).

## Discussion

*Branchinecta lynchi* is phylogeographically structured at a regional scale across the species range and shows a pattern of historical isolation correlated with the geographic boundaries of isolated watersheds corresponding to vernal pool regions identified in the Recovery Plan for the species. This genetic structuring across the species range suggests that long distance dispersal resulting in gene flow by wind or waterfowl is not common among populations of this species. Among the 104 COI haplotypes sampled across the species’ range, there were only two instances of a shared haplotype between vernal pool regions, indicating either a long-distance dispersal event between these pairs of vernal pool regions or retention of shared ancestral polymorphism. Importantly, our data (Model 4, Fig 2) show a high degree of isolation even among sampling localities within vernal pool regions as indicated by the high Φ_ct_, of which is similar to that observed for other fairy shrimp species [11, 21]. Because vernal pools within regions can vary in soil and water chemistry and inundation timing over short distances [48, 49] [50], additional adaptive variation may exist within the broad geographic regions examined here. As with many studies examining listed species [51], our sampling efforts were limited to prevent adverse impacts to populations. Consequently, we were not able to examine fine-scale patterns associated with soils, local hydrology, or local anthropogenic activities. To the degree we could test this with isolation by distance (Fig 6), some vernal pool regions exhibit isolation by distance patterns. Additional neutral and adaptive variation may be associated with the ecological differences among pools within regions as well as human-caused vicariant events due to habitat loss resulting from development and non-compatible agricultural practices. However, patterns of isolation by distance and local adaptation will need to be confirmed with sampling at finer scales.

The low gene flow among regions assessed in this study presents an interesting conservation challenge for fairy shrimp in general. For *B*. *lynchi* in particular, the extant range is quite large spanning more than ten degrees of latitude and the species currently exists in isolated patches throughout its remaining range. The high degree of genetic endemism in COI sequences across this range suggests that dispersal resulting from interbreeding among regions rarely occurred at least on historical time scales. Dispersal limitation at local scales is supported by the strong pattern of isolation by distance and this mechanism, along with possible adaptation to local environmental conditions, is a likely hypothesis that would explain population genetic structuring within, but not among vernal pool regions, where long-term isolation and genetic drift or regional patterns of adaptation are likely stronger forces driving genetic structure across the species range [52].

Because much of the genetic diversity of *B*. *lynchi* is unique and isolated, planning and implementing translocations may be challenging. To broadly maintain extant neutral genetic diversity among vernal pool regions, as well as adaptive variation that might be present, translocations and reintroductions should proceed after the demographic benefits of translocation have been weighed against the potential for disruption of remaining historical phylogeographic structure. However, managers who wish to implement translocations should consider that it is possible our estimates of historical gene flow and diversity of *B*. *lynchi* are incomplete because we had small sample sizes from some vernal pool regions (WRVP, SJVP and CCVP) and because our samples are from post habitat reduction; thus, not allowing a full analysis across the historic species range. Unfortunately, due to preservation techniques (e.g., use of formalin), and as our data indicate (Fig 3), museum specimens are not likely to easily yield genetic data from specimens sampled from locations where the habitat has been lost. Thus, future genetic work with the species should focus on non-lethal sampling techniques sourcing DNA from sediment or water to complete our knowledge of extant populations and such work may provide valuable insights prior to implementation of translocations among vernal pool throughout the range of the species.

A promising area of further conservation research is analysis of sediment cores or water samples for environmental DNA (eDNA) [32, 53]. This species, with its resting egg state, would be an ideal species for which to test the utility of eDNA from sediment cores in vernal pools. Additionally, extracted DNA from water samples provides a non-lethal sampling tool for assessment of presence when the species cannot be directly observed or for populations where sampling of adult individuals may cause an unacceptable level of risk to the persistence of the local population [54]. An eDNA approach may provide an alternative to traditional dipnet surveys and contribute new information about current and potentially historical range of genetic diversity for the species. For example, because *B*. *lynchi* is sparsely distributed across its extant range, knowledge of whether the species is present within specific vernal pool complex is often lacking. A more thorough description of its fine-scale distribution with a tool such as eDNA techniques would help identify important habitat linkages, provide occupancy data, and could be used to inform translocation efforts.

We found evidence of a potential long distance dispersal event between the SSFH and KMVP regions. While the haplotype sampled in KMVP region is similar to those sampled in SSFH region, it was not identical, indicating that it has diverged since dispersal occurred, or that the haplotype found in KMVP was not sampled from SSFH in our study. This long distance dispersal event was also detected in a previous study [26]; however, the genetic material used for the Aguilar 2011 analysis came from the same specimen lot as ours from the California Academy of Sciences museum collection (CAS 172319). The most likely mechanisms for this dispersal event are either waterfowl or human-facilitated translocation. Waterfowl are known dispersal vectors for branchiopods [16] and appear to be capable of moving sufficient numbers of cysts to homogenize branchiopod populations under certain circumstances [24, 25]. Among the *B*. *lynchi* populations sampled here, we observed potential long-distance dispersal only once across the species range. This, in combination with the phylogeographic signal recovered among vernal pool regions, suggests that long-distance dispersal of *B*. *lynchi* by birds, or another mechanism, is not likely to be a common event.

The reduction in informative sites by using the partial barcode for COI likely led to the previous conclusion of weak phylogeographic structure for this species [26]; in contrast, through analysis of the full length sequence for the COI barcode region we find evidence of phylogeographic structure across the species’ range. Analysis of the 16S gene, however, revealed very little population structure across the species range. The low genetic diversity within the species for this gene compared to that of the high genetic divergence with other *Branchinecta* species used as outgroups, supports the monophyly of the species throughout its patchy range. The 16S gene is known to evolve at a slower rate compared to COI in crustaceans [55] and is a likely explanation for why the two genes display such drastically different signals of phylogeographic structure. By contrasting the 16S and COI datasets we recovered support for both monophyly of the species across its large geographic range and support for population genetic structure associated with many of the vernal pool regions established in the recovery plan for the species. Future efforts to create a molecular phylogenetic tree for this genus and family are needed, possibly employing next-generation sequencing, before more detailed conclusions can be drawn about the divergence of this species from other endemic *Branchinecta* species in California.

Use of historical samples from museum specimens proved quite challenging in this study. Unfortunately for many specimens collected prior to 2000 the original fixative was not recorded and they are now only stored at 75% ETOH in the museum collection. We suspect any future studies using museum specimens from before 2000 will require significant time and investment to yield useful genetic data. A recent study testing known preservation methods used for Anostracan species, including *B*. *lynchi*, found that use of non-denatured ethanol likely contributed to the decreased value of museum collections for future DNA studies [56]. Given the conservation status for the species we highly recommend storage of specimens collected during any monitoring activities and programs in molecular grade 95% ethanol to increase their value for any potential genetic studies in the future.

## Conclusions

The molecular evidence supports, at a minimum, considering each vernal pool region as a separate genetic management unit [9]. Previous research in other branchiopod species has shown that additional population genetic structure may be present at even finer scales [57]. At a regional-scale, the phylogenetic patterns identified here can assist in conservation reserve design and help determine areas harboring unique genetic diversity that may be prioritized for restoration or conservation protection for the species. A thorough investigation of local adaptations and species habitat requirements will provide additional critical insights that are needed before long-distance translocations are contemplated. A genetic management plan could help to provide specific guidance on potential source and donor populations and the appropriate geographic scale for translocations.

## Supporting information

S1 Fig Phylogenetic support for nodes estimated from a bootstrap analysis of the tree depicted in Fig 3. The geographic sample information for each number associated with each haplotype is given in Table 1.
(PDF)

S2 Fig Phylogenetic support for nodes estimated from a Baysian posterior probability analysis of the tree depicted in Fig 3. The geographic sample information for each number associated with each haplotype is given in Table 1.
(PDF)

S3 Fig Mismatch distributins plotted for each vernal pool region. Solid line depicts expected pattern under a constant population size and the dashed line indicates observed values. Samples from the San Joaquin Valley (SJVP) vernal pool region was excluded because the sample size was to small for estimating the distribution.
(PDF)

S4 Fig Reduced median joining network of haplotyes based on 16S gene sequences obtained from *B*. *lynchi* throughout its range. Open circles in network are hypothesized, but not sampled haplotypes. Abbreviations are as in Table 1.
(TIF)

S1 Table Metadata associated with each specimen analyzed in this study. Table includes the geographic location in latitude and longitude, the vernal pool region for each site sampled, the haplotype name as given in Fig 3 for COI and for 16S as in Table 1 and the accession numbers for each sequence in GenBank.
(XLSX)
