# Supplementary figures and images for: Range-wide phylogeographic structure of the vernal pool fairy shrimp (Branchinecta lynchi)
Source: PLoS One. 2017 May 4;12(5):e0176266. doi: 10.1371/journal.pone.0176266 (PMC5417434; doi:10.1371/journal.pone.0176266)

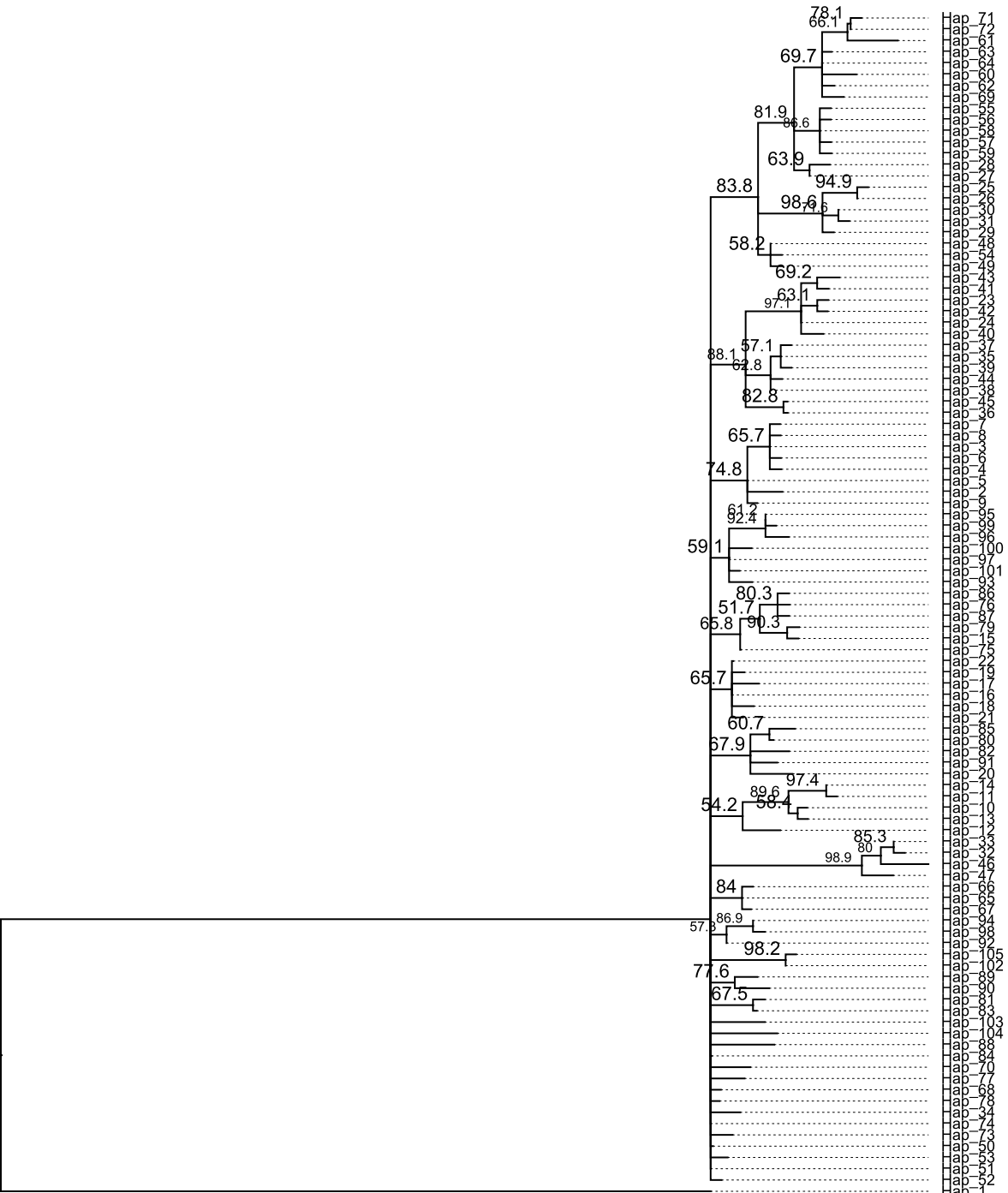

0.02

Supplement: S1 Fig — The geographic sample information for each number associated with each haplotype is given in Table 1. (PDF) [file pone.0176266.s001.pdf]

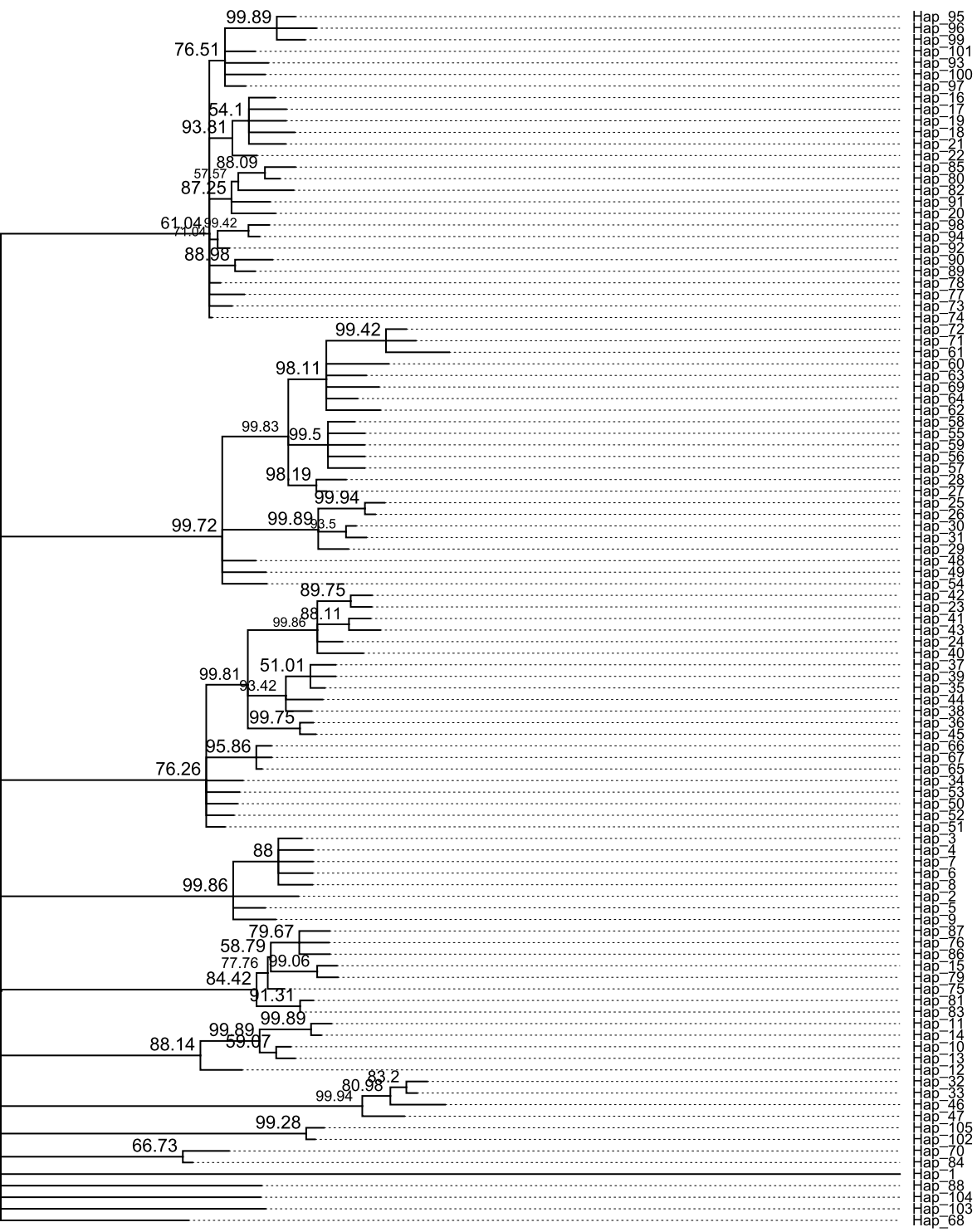

0.3

Supplement: S2 Fig — The geographic sample information for each number associated with each haplotype is given in Table 1. (PDF) [file pone.0176266.s002.pdf]

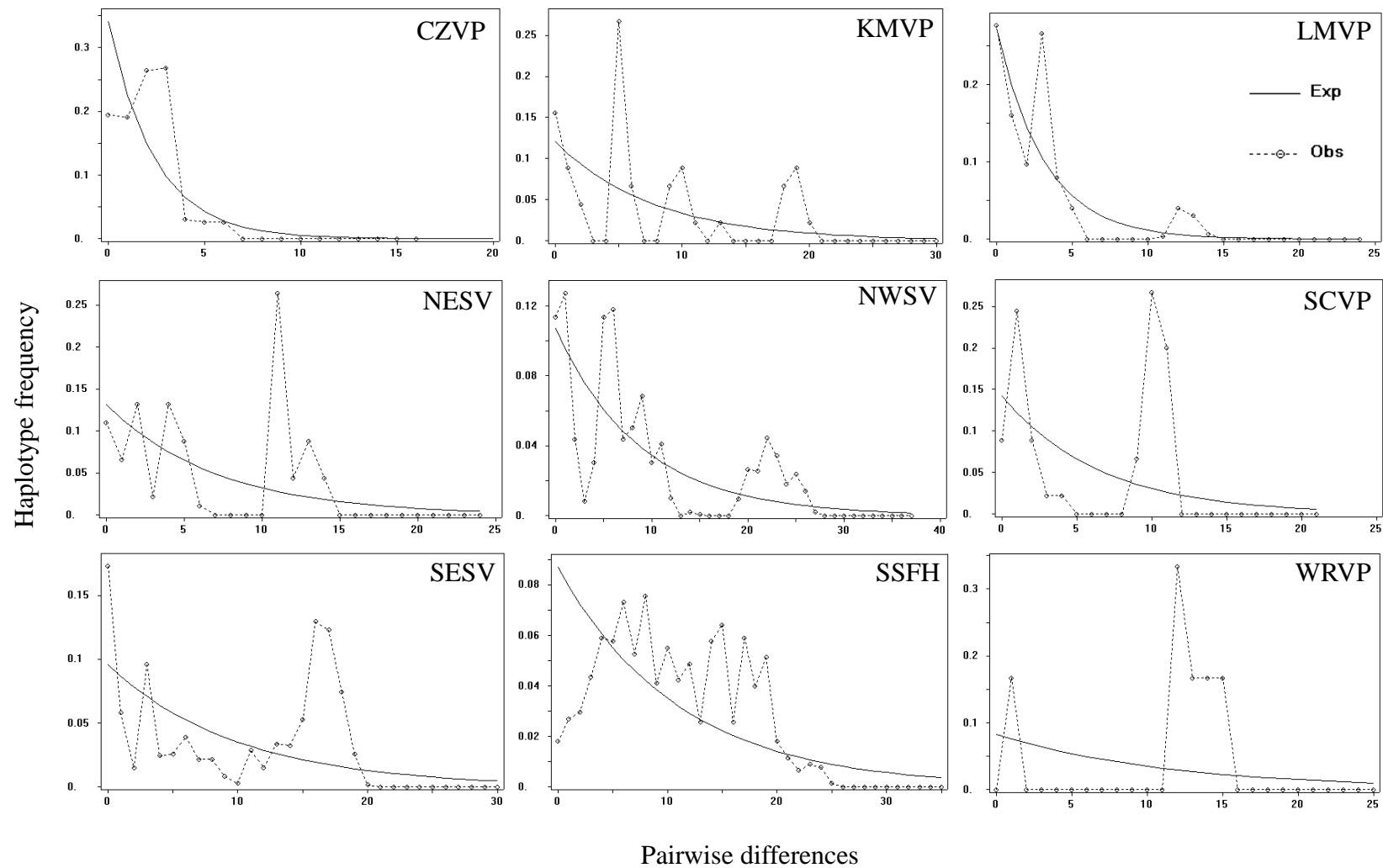

Supplement: S3 Fig — Solid line depicts expected pattern under a constant population size and the dashed line indicates observed values. Samples from the San Joaquin Valley (SJVP) vernal pool region was excluded because the sample size was to small for estimating the distribution. (PDF) [file pone.0176266.s003.pdf]

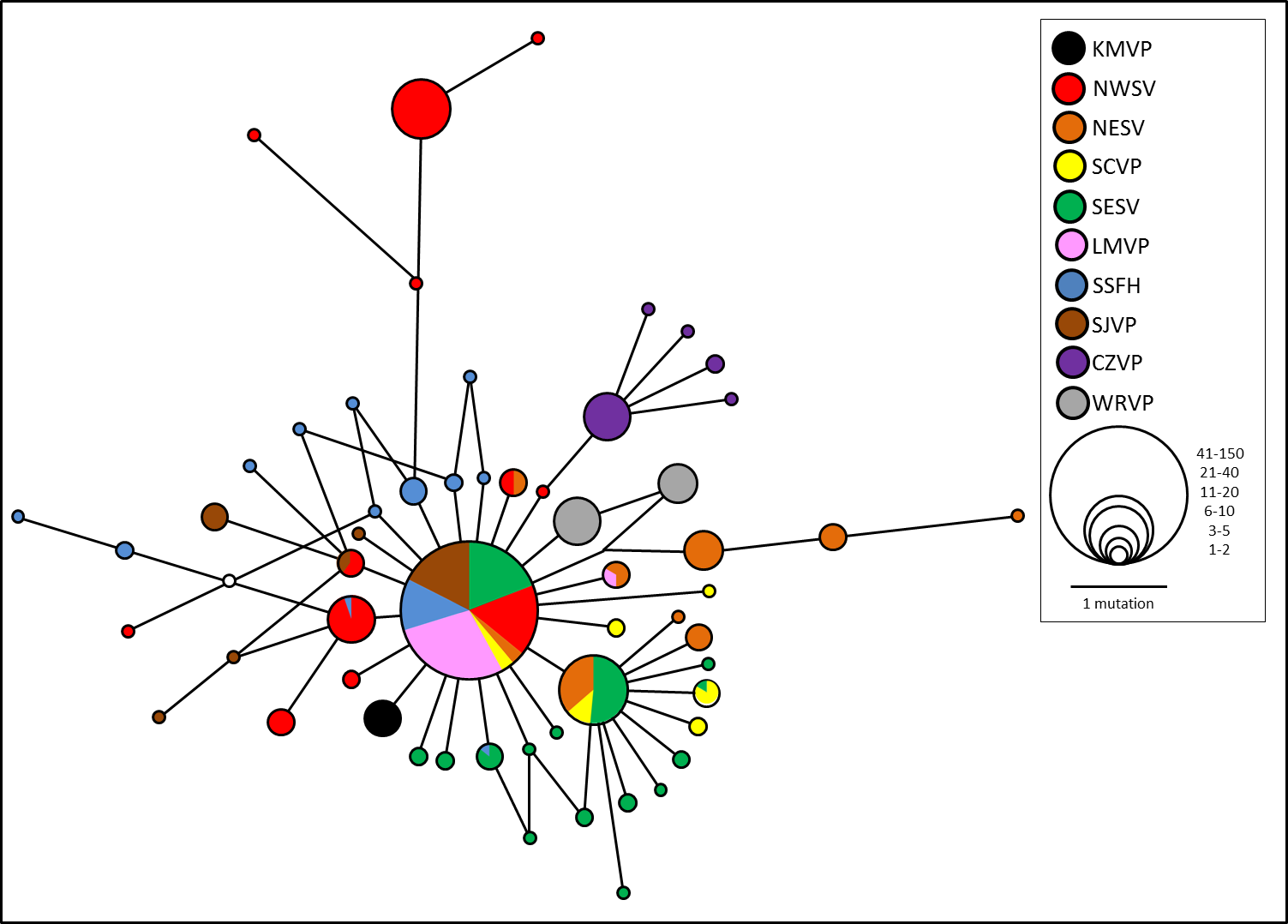

Supplement: S4 Fig — Open circles in network are hypothesized, but not sampled haplotypes. Abbreviations are as in Table 1. (TIF) [file pone.0176266.s004.tif]
